# Unmet Needs in the Diagnosis and Management of Early AD in Community‐Based Settings in the United States

**DOI:** 10.1002/alz.089135

**Published:** 2025-01-09

**Authors:** Timothy R Juday, Ashley A Holub, Keith A. Betts, Soeren Mattke, Sophie A Kitchen, Hongjiao Liu, Amyn Malik, Richard Batrla, Feride H Frech, Ara S Khachaturian

**Affiliations:** ^1^ Eisai Inc., Nutley, NJ USA; ^2^ Analysis Group, Boston, MA USA; ^3^ Analysis Group, Los Angeles, CA USA; ^4^ University of Southern California, Los Angeles, CA USA; ^5^ Prevent Alzheimer’s Disease 2020, Inc., Rockville, MD USA

## Abstract

**Background:**

Disparities in the quality and timeliness of care for Alzheimer’s disease (AD) are well documented. This study assessed the impact of demographic characteristics on the diagnosis and management of early AD patients in community‐based settings.

**Method:**

This cross‐sectional study abstracted medical chart data for patients aged 50‐89 years who had newly diagnosed early AD (mild cognitive impairment [MCI] or mild AD) within the past 2 years and a clinic visit within the past 12 months. Data collected included use of neurocognitive assessments, imaging/biomarker tests, laboratory tests, referrals, and treatments. Descriptive statistics were stratified by patient age at diagnosis, sex, race, and residence.

**Result:**

A total of 1284 early AD patients (817 MCI/467 mild AD) had a mean age at diagnosis of 70 years, 43% were female, 31% were non‐white, and 17% were rural. The number of visits from initial complaint to diagnosis was lower (2.6 vs 3.0, p<0.001), and time to referral consult was faster (20.9% vs 54.4% waited ≥4 months, p<0.001) for patients ≥65 relative to <65 years. Biomarker/imaging tests were also more common (78.2% vs 72.4%, p<0.05), and beta‐amyloid medications were less frequently prescribed (3.0% vs 15.6%, p<0.001). Practices were similar for female and male patients. Non‐white patients had a longer time to diagnosis (5.5 vs 4.6 months, p<0.01) and were less likely to receive any neurocognitive assessments (80.1% vs 85.5%, p<0.05) than white patients. APOE‐e4 testing was more often used in non‐white patients (20.9% vs 17.5%, p<0.01). Urban/suburban patients were more likely to receive the ADAS‐Cog (19.2% vs 11.6%, p<0.01), while rural patients were more likely to receive the MoCA (28.2% vs 18.9%, p<0.01). Rural patients were less likely to be prescribed beta‐amyloid medications (2.3% vs. 6.5%) and more likely to wait at least 4 months for a prescription (80.0% vs.45.6%).

**Conclusion:**

Initiatives that shorten the time to diagnosis and to referral consults in patients aged <65, and that promote the use of neurocognitive assessments in non‐white patients may help close the gap. As early detection and treatment are critical to optimize patient outcomes, more effort needs to be focused on reducing care disparities.

